# Colonization and decolonization of global health: which way forward?

**DOI:** 10.1080/16549716.2023.2186575

**Published:** 2023-03-20

**Authors:** Maysoon Hussain, Mitra Sadigh, Majid Sadigh, Asghar Rastegar, Nelson Sewankambo

**Affiliations:** a Ross University School of Medicine, Miramar, FL, USA; b Stony Brook University Renaissance School of Medicine, Stony Brook, NY, USA; c Department of Education and Innovation, Nuvance Health/University of Vermont College of Medicine, Burlington, NJ, USA; d Department of Medicine, Yale School of Medicine, New Haven, CT, USA; e Makerere College of Health Sciences, Makerere University School of Medicine, Kampala, Uganda

**Keywords:** Colonisation, decolonisation, global health education, high-income countries, low-income countries

## Abstract

Despite taking on several forms throughout history such as colonial medicine, tropical medicine, and international health, the field of global health continues to uphold colonialist structures. History demonstrates that acts of colonialism inevitably lead to negative health outcomes. Colonial powers promoted medical advancements when diseases affected their own people, and only did so for locals when in the colonies’ best interests. Numerous medical advancements in the United States also relied on the exploitation of vulnerable populations. This history is critical in evaluating the actions of the United States as a proclaimed leader in global health. A significant barrier to progress in the field of global health is that most leaders and leading institutions are located in high-income countries, thereby defining the global standard. This standard fails to meet the needs of most of the world. In times of crisis, such as the COVID-19 pandemic, colonial mentalities may be more evident. In fact, global health partnerships themselves are often ingrained in colonialism and may be counterproductive. Strategies for change have been called into question by the recent Black Lives Matter movement, particularly in evaluating the role that less privileged communities should have in their own fate. Globally, we can commit to evaluating our own biases and learning from one another.

## A brief history of colonisation in global health

Despite being given several names throughout history, from its roots in colonial medicine [1] to tropical medicine, international health, and even planetary health, the field of global health continues to uphold colonialist structures. From its use as a scientific rationale for assigning racial superiority or inferiority to its restrictions on the movement of indigenous people in the name of infection control, the exploitation of local remedies, to the injustices against people of colour in the name of medical progress, stories of inequity have long been woven into the history of global health [2]. Every act of colonialism, be it enlisting of indigenous populations into hard labour, usurping of ancient culture and faith practices, or the imposition of divisive borders, has inevitably led to negative health outcomes [3].

French colonial strategist Herbert Lyautey described medicine as the ‘most effective of our agents for penetration and pacification’ [4]. History demonstrates that colonial powers promoted medical research and advancements when diseases affected their own people, and only did so for locals when in the colonies’ best interests, such as the case of malaria control in the construction of the Panama Canal [5]. In fact, in the United States, there are many examples of medical advancements that relied on exploitation of vulnerable populations. The vesicovaginal fistula surgery was crafted by Marion Sims who became known as the ‘father of modern gynecology’ without any credit given to the enslaved black women who were operated on without anaesthesia [6]. The development of chemotherapy by Cornelius Rhoads earned him countless accolades while the 60,000 US soldiers of African, Japanese, and Puerto Rican descent who suffered lifelong consequences of his mustard gas experiments went unnamed. The Tuskegee Study, the Guatemala Syphilis Study [7,8], forced sterilisations in Virginia [9], and the Stateville Penitentiary Malaria Experiments [10] among countless others evidence the nation’s long and gruelling medical history. Even in modern times, forced sterilisations and separation of families continue in border communities [11]. This history is critical in evaluating the actions of the United States as a proclaimed leader in global health.

## Barriers to progress

With scientific leaders, leading institutions, medical journals, and authors primarily located in high-income countries (HICs), their medical practice and points of view stand as the global standard for all [12,13]. Rather than developing standards based on their own unique settings, communities in low- and middle-income countries (LMICs) strive and often struggle to adhere to practices that are not easily applicable to their context. Diseases of poverty are ignored when afflicted populations are unable to produce economic profits for those who control medical markets. In short, health ideals created by HICs consistently fail to consider the needs of most of the world, and communities that are already suffering due to coloniality-caused power and other imbalances suffer even more. The world witnessed how ‘handwashing, social distancing, and lockdowns’ protocols disseminated by HICs in response to the COVID-19 pandemic failed in areas where water is scarce, lockdowns sacrifice livelihoods, and people are crowded into small homes or refugee camps – which are sequelae of coloniality [2].

Vulnerable communities are also subject to scapegoating in times of crisis. This has been prevalent throughout history and seen yet again with the labelling of ‘Chinese Virus’ or ‘Wuhan Virus’ and the surge of hate crimes directed at Asian Americans [14]. Meanwhile, French physicians Camille Locht and Jean-Paul Mira suggested that vaccine trials should be carried out in Africa ‘where people are highly exposed and don’t protect themselves,’ [15] language that is directly colonial. In fact, some LMICs had effective COVID-19 response strategies such as lockdowns and test-and-trace schemes in Kerala, and the use of innovative technologies for affordable tests in Senegal [16] but their advantages did not gain as much attention as their drawbacks.

Limited within other colonial structures, global health partnerships that aim to ‘help’ often mirror colonial relationships, with members of HICs being given greater opportunities (mentorship, employment opportunities, leadership positions, compensation) in LMICs than the other way around [17]. And yet, it is unclear what reciprocity should look like in terms of benefits to LMIC communities, as global health programmes are also known to amplify ‘brain drain’ rather than nurture ‘brain gain.’ Meanwhile, global health participants travelling from HICs to LMICs often become more deeply ingrained in the colonial mentality as they are not encouraged to investigate the causes of the inequities they witness. In carrying forward the legacy of white supremacy, partners from HICs tend to share knowledge without reciprocating with an openness to learning themselves.

## Next steps

The Black Lives Matter movement has recently invoked discussion about effective strategies to produce meaningful change. Dr Liboa Hirsch, a Research Fellow at the London School of Hygiene and Tropical Medicine, explores whether it is possible to achieve decolonisation through the same structures that were built to uphold colonialism and white supremacy [18]. While she does not advocate for violence, she demands ‘anger and revolution’ to dismantle such systems, stating that Black, Indigenous, and people of colour do not need to be ‘set free’ as they will continue to fight for their own freedom. When applied to the field of global health, perhaps people from HICs should question their role altogether. Should they be engaging affected populations towards demanding change? Should they be providing resources for groups to rise up and demand change? Or should they simply step aside so that these communities can be heard more loudly? Perhaps, we should ask those in LMICs what supportive role, if any, HICs should play in their struggle – a question that should always be at the centre but seems to be seldom asked.

The BMJ Global Health suggests a paradigm shift that would involve actively acknowledging oppressive forces; leadership shift, to include more women, minorities, and experts from LMICs; and a knowledge shift from unidirectional to bidirectional flow between LMICs and HICs [2]. Global Intellectual History [19] suggests a comparative method in knowledge exchange. The focus on comparison innately implies equality. They also endorse focusing on ‘reconstruction’ of Global Health, rather than its deconstruction, or decolonisation. In a practical sense, accomplishing these changes in mentality and knowledge shifts would require active participation by both LMICs and HICs. In global health, no one should be left behind.

Meaningful change cannot occur within systems designed to uphold colonial and neo-colonial powers. In the powerful words of activist Audre Lorde, ‘The Master’s tools will never dismantle the Master’s house.’ Global health institutions in HICs would need to transfer power back to LMICs and face strict limitations on their ability to intervene in communities that have been overtaken for too long. This is not feasible when HICs want to neither cede that power nor accept the push of LMICs for greater autonomy. The loudest voices in the decolonisation movement are those of HICs threatening to turn meaningful agendum into an echo chamber that paradoxically pushes out the voices that, according to the movement’s doctrine, need to lead.

We must acknowledge the limitations of changing a system that is not only itself rooted in coloniality but is entrenched within other colonially rooted systems. We must acknowledge the inevitable futility of this task as long as HICs continue to define the narrative, the standards, the distribution of resources, and the direction of the decolonisation movement. We must acknowledge the ways HICs contradict their professed global health agenda by critically evaluating power structures within their own nations. Globally, we can commit to increasing our awareness of longstanding inequities by becoming actively anti-supremacist, anti-oppressionist, and anti-racist within our own biases, and integrating these values into medical education. We must accept that in ‘decolonising’ or ‘deconstructing’ [19], priviledged populations may have more to learn and less to teach.

## Paper context

Global health is rooted in colonialism. Throughout history and its many forms, its colonialist structures have been upheld. Efforts to decolonise global health have varied in effectiveness. This paper examines the roots of global health, recent developments in the decolonisation of global health, barriers to progress, and our proposals for the next steps forward.

## References

[1] Abimbola S, Pai M. Will global health survive its decolonisation? *Lancet* 2020;396:1627–3. 33220735 10.1016/S0140-6736(20)32417-X

[2] Büyüm AM, Kenney C, Koris A, Mkumba L, Raveendran Y. Decolonising global health: if not now, when? J Obstet Gynaecol. 2020;5:e003394. 10.1136/bmjgh-2020-003394 PMC7409954 32759186

[3] Klein C. Why the construction of the Panama Canal was so difficult—and deadly. History. [cited 2021 Oct 25]. Available from: https://www.history.com/news/panama-canal-construction-dangers

[4] Eichbaum Q, Adams LV, Evert J, Ho M, Semali IA, Van Schalkwyk SC. Decolonizing Global Health Education: Rethinking Institutional Partnerships and Approaches. Acad Med. 2021 Mar 1;96:329–335. 32349015 10.1097/ACM.0000000000003473

[5] Tilley H. Medicine, empires, and ethics in colonial Africa. AMA J Ethics. 2016;18:743–753. 27437825 10.1001/journalofethics.2016.18.7.mhst1-1607

[6] Wall LL. The medical ethics of Dr. J Marion Sims: a fresh look at the historical record. J Med Ethics. 2006 Jun;32:346–350. 16731734 10.1136/jme.2005.012559 PMC2563360

[7] Campbell ND, Stark L. Making up ‘vulnerable’ people: human subjects and the subjective experience of medical experiment. Soc History Med. 2015 Nov 1;28:825–848.

[8] Reverby SM. Ethical failures and history lessons: the U.S. Public Health Service research studies in Tuskegee and Guatemala. Public Health Rev. 2012;34:13. 10.1111/bioe.12784 32608027

[9] Rowlands S, Amy J-J. Sterilization of those with intellectual disability: evolution from non-consensual interventions to strict safeguards. J Intellect Disabil. 2019;23:233–249. 29228865 10.1177/1744629517747162

[10] Comfort N. The prisoner as model organism: malaria research at Stateville Penitentiary. Stud Hist Philos Biol Biomed Sci. 2009 Sep;40:190–203. 19720327 10.1016/j.shpsc.2009.06.007 PMC2789481

[11] Re: Lack of medical care, unsafe work practices, and absence of adequate protection against COVID-19 for detained immigrants and employees alike at the Irwin County Detention Center. Project South: Institute for the Elimination of Poverty & Genocide. [cited 2020 Sep 14]. Available from: https://projectsouth.org/wp-content/uploads/2020/09/OIG-ICDC-Complaint-1.pdf

[12] Mbaye R, Gebeyehu R, Hossmann S, Mbarga N, Bih-Neh E, Eteki L, et al. Who is telling the story? A systematic review of authorship for infectious disease research conducted in Africa, 1980–2016. BMJ Global Health. 2019;4:e001855. 10.1136/bmjgh-2019-001855 PMC6830283 31750001

[13] Bile K, Emmelin M, Freij L, Gustafsson LL, Sahlén K, Wall S. Who published what on Somali health issues. Somali Health Action J. 2022;2. DOI:10.36368/shaj.v2i1.281

[14] Rogin A, Nawaz A. ‘We have been through this before.’ Why Anti-Asian hate crimes are rising amid coronavirus. PBS; [cited 2020 Jun 25]. Available from: https://www.pbs.org/newshour/nation/we-have-been-through-this-before-whyanti-asian-hate-crimes-are-rising-amid-coronavirus

[15] Rossman R. Racism row as French doctors suggest virus vaccine test in Africa. Al Jazeera; [cited 2020 Apr 4]. Available from: https://www.aljazeera.com/news/2020/4/4/racism-row-as-french-doctors-suggest-virus-vaccine-test-in-africa

[16] Prajitha KC, Rahul A, Chintha S, Soumya G, Suresh MM, Nair ANKK, et al. Strategies and challenges in Kerala’s response to the initial phase of COVID-19 pandemic: a qualitative descriptive study. BMJ Open. 2021;11:e051410. 10.1136/bmjopen-2021-051410 PMC8275365 34244285

[17] Kulesa J, Brantuo NA. Barriers to decolonising educational partnerships in global health. BMJ Global Health. 2021;6:e006964. 10.1136/bmjgh-2021-006964 PMC8601064 34789513

[18] Hirsch LA. Is it possible to decolonise global health institutions? Lancet 2021;397:189–190. 33453772 10.1016/S0140-6736(20)32763-X

[19] Herbjørnsrud D, Herbjørnsrud D. Beyond decolonizing: global intellectual history and reconstruction of a comparative method. Global Intellect History 2019;6:1–27.

